# Stathmin overexpression is associated with growth, invasion and metastasis of lung adenocarcinoma

**DOI:** 10.18632/oncotarget.11006

**Published:** 2016-08-02

**Authors:** Lin Yurong, Rong Biaoxue, Li Wei, Ming Zongjuan, Shi Hongyang, Fang Ping, Gao Wenlong, Yang Shuanying, Li Zongfang

**Affiliations:** ^1^ Department of Respiratory Medicine, Second Affiliated Hospital, Xi'an Jiaotong University, Xi'an, China; ^2^ Department of Statistics and Epidemiology, Medical College, Lanzhou University, Lanzhou, China; ^3^ Department of Elderly Surgery, Second Affiliated Hospital, Xi'an Jiaotong University, Xi'an, China

**Keywords:** stathmin, lung adenocarcinoma, shRNA, tumor growth, proliferation

## Abstract

Stathmin has been investigated as a tumor biomarker because it appear to be associated with tumorigenesis; however, the effect of stathmin in lung adenocarcinoma (LAC) remains poorly understood. The purpose of this study was to examine the expression of stathmin in lung adenocarcinoma, and to disclose the relationship between them. The expression of stathmin was examined by RT-PCR, IHC and Western blot. Furthermore, small interfering RNA (shRNA)-mediated silencing of stathmin was employed in LAC cells to investigate cell proliferation, invasion and apoptosis. In this study, we showed that overexpression of stathmin was significantly associated with poorly differentiated, lymph node metastasis and advance TNM stages of lung adenocarcinoma. And silencing of stathmin expression inhibited the proliferation, migration and invasion of lung adenocarcinoma PC-9 cells, and retarded the growth of PC-9 cells xenografts in nude mice. Additionally, the anticarcinogenic efficacy of stathmin silencing might be involved in P38 and MMP2 signaling pathways. In conclusion, these results showed that stathmin expression was significantly up-regulated in LAC, which may act as a biomarker for LAC. Furthermore, silence of stathmin inhibiting LAC cell growth indicated that stathmin may be a promising molecular target for LAC therapy.

## INTRODUCTION

Lung cancer is a major public health problem in China, which has a high mortality and a high morbidity. It is reported that there will be about 733,000 newly diagnosed invasive lung cancer cases in 2015 in China, and about 610,000 Chinese will die from lung cancer in 2015 [1]. The main reason for higher mortality is that most cases of lung cancer are diagnosed at an advanced stage. Nowadays, researchers agree that early diagnosis and individualized therapy are very important to improve survival and prognosis. Thus, development of new technology on diagnosis and therapy are greatly needed.

Stathmin is composed of 149 amino acids, which are organized into four domains (I–IV). The core region of stathmin (amino acids 42–126) is site for tubulin interaction with the additional requirement of either an N- or C-terminal extension [2]. Previous studies suggest that stathmin is overexpressed in some types of human malignancies [3–8]. And, high expression of stathmin can potentially promote cell proliferation, motility and metastasis of malignant tumors [4, 9–11]. However, the study on the expression of stathmin and its clinical significance in lung adenocarcinoma (LAC) is considerably limited. The aim of this study is to investigate the expression of stathmin in lung adenocarcinoma and evaluate the anticarcinogenic effects of stathmin silencing.

## RESULTS

### Expression of stathmin in cancer and normal tissues

**Figure 1:**
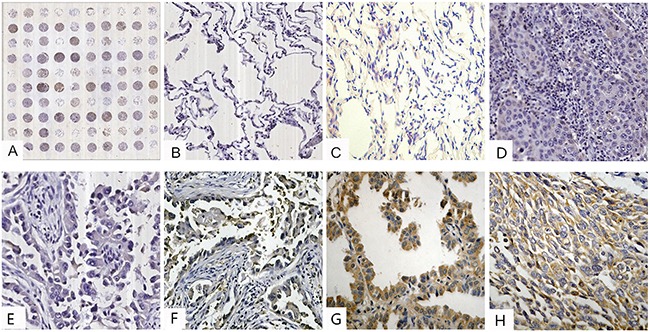
IHC analysis of stathmin in lung cancer and normal lung tissues (IHC×400) **A**. tissue microarray construction; **B**. low staining of stathmin in normal lung tissues; **C**. moderate staining of stathmin in normal lung tissues; **D**. low staining of stathmin in well differentiated LSCC; **E**. low staining of stathmin in well differentiated LAC; **F**. moderate staining of stathmin in moderately differentiated LAC; **G**. high staining of stathmin in poorly differentiated LAC; **H**. moderate staining of stathmin in moderately differentiated LSCC; LAC, lung adenocarcinoma; LSCC, lung squamous cell carcinoma.

**Figure 2:**
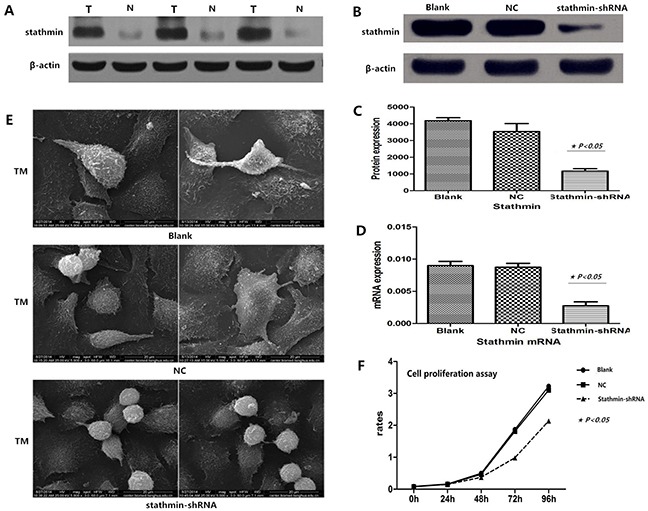
Efficacy of stathmin-shRNA in lung adenocarcinoma cells **A**. western blot showed that stathmin was overexpressed in LAC tissues compared with normal lung tissues (*p*<0.05); **B-C**. western blot showed stathmin-specific shRNA decreased the expression of stathmin (*p*<0.05); **D**. real-time PCR showed that stathmin-specific shRNA decreased the expression of stathmin mRNA (*p*<0.05); **E**. transmission electron microscopy showed that stathmin-specific shRNA weakened the deformability of PC-9 cells and reduced pseudopodia of PC-9 cells, as compared to the blank and NC groups; **F**. proliferation of PC-9 cells transfected with stathmin-shRNA was significantly decreased, as compared with the blank and NC groups; NC, lung adenocarcinoma PC-9 cells transfected with negative control shRNA; TEM, transmission electron microscopy.

Stathmin was expressed in the cellular protoplasm, which was colored brown by immunohistochemistry (IHC) (Figures 1A, B, C, D, E, F, G, and 1H). Stathmin was highly expressed in 31 (38.7%) of the 80 lung cancer tissues, whereas was lowly expressed in 12 (15%) of the 80 normal tissues (p<0.05) (Table 1). To further confirm these findings, the expression level of stathmin was detected in paired LAC tissue specimens by Western blot. As shown in Figure 2A, stathmin was overexpressed in cancerous tissues compared with normal tissues (p<0.05).

**Table 1:** Correlation between clinico-pathological features and expressions of stathmin in lung cancer

Parameter	Group	N	Expression of Stathmin				
			Low (%)	Moderate (%)	High (%)	χ^2^ value	*P* value
Item							
	Normal	80	50(62.5)	18(22.5)	12(15)^®^	21.084	**0.001**
	Cancerous	80	22(27.5)	27(33.8)	31(38.7)		
Gender							
	Male	66	18(27.3)	22(33.3)	26(39.4)	0.067	0.967
	Female	14	4(28.6)	5(35.7)	5(35.7)		
Ages							
	<60	42	10(23.8)	16(38.1)	16(38.1)	0.984	0.624
	≥60	38	12(31.6)	11(28.9)	15(39.5)		
Smoking							
	0	38	12(31.6)	14(36.8)	12(31.6)	0.696	0.792
	0.1–40	12	3(25)	4(41.7)	5(33.3)		
	>40	30	7(23.3)	9(30)	14(46.7)		
Histology							
	LAC	38	4(10.5)	12(31.6)	22(57.9) ^▲^	14.53	**0.001**
	LSCC	42	18(42.9)	15(35.7)	9(21.4)		
Pathological Grade							
	Poorly	29	5(17.2)	6(20.7)	18(62.1)^★^	16.351	**0.003**
	Moderately	30	7(23.3)	16(53.3)	7(23.3)		
	Well	21	10(47.6)	5(23.8)	6(28.6)		
Lymphatic Invasion							
	N0	52	19(36.5)	20(38.5)	13(25.0)■	15.279	**0.018**
	N1	15	2(13.3)	5(33.3)	8(53.3)		
	N2	10	1(10)	2(20)	7(70)		
	N3	3	0(0)	0(0)	3(100)		
pTNM							
	IB	3	0(0)	3(100)	0(0) ^●^	15.194	**0.019**
	IIA-IIB	33	14(42.4)	11(33.3)	8(24.2) ^●^		
	IIIA-IIIB	40	8(20)	12(30)	20(50)		
	IV	4	0(0)	1(25)	3(75)		

^®^*p*=0.001, normal tissues compared with cancerous tissues;^▲^*p*=0.001, LAC compared with LSCC; ^★^*p*=0.003, poorly differentiated tissues compared with moderately and well differentiated tissues; ^■^*p*=0.018, N0 compared with N1, N2, and N3, respectively; ^●^*p*=0.019, IB and IIA-IIB compared with IIIA-IIIB and IV, respectively; LAC, lung adenocarcinoma; LSCC, lung squamous cell carcinoma; Smoking, pack years of smoking.

### Correlation between expression of stathmin and clinicopathologic factors of lung cancer

Stathmin was overexpressed in lung adenocarcinoma (LAC) (22/38, 57.9%) compared with lung squamous cell carcinoma (LSCC) (9/42, 21.4%) (p<0.05). Poorly differentiated lung cancer tissues displayed a higher expression of stathmin (62.1%) than well-differentiated tissues (28.6%) (p<0.05). The expression of stathmin in cancer tissues without lymph node metastasis was 25%, which is lower than those cases with lymphatic invasion as follows: N1, 53.3%; N2, 70%; and N3, 100%, respectively (p<0.05). And increased stathmin was also observed in cancer tissues with stages III (68%) and IV (75%), compared with cases of stages I (both 0%) and II (24.2%) (p<0.05) (Table 1).

### Silencing of stathmin inhibited expression of stathmin

Lung adenocarcinoma PC-9 cells were transfected by two different shRNAs (shRNA1 and shRNA2) targeting the stathmin gene. After 48 h, we harvested the cells and detected the protein and mRNA levels of stathmin using RT-PCR and Western blot. Relative quantification analysis revealed that stathmin- specific shRNA (shRNA1) significantly down-regulated the expression of protein and mRNA of stathmin (p<0.05) (Figure 2B, C and 2D). Therefore, the PC-9 cells that were transfected with shRNA1 (shRNA) was used to carry out following experiments. The following experiments were divided into three groups: blank, negative control (NC, non-specific to any known gene), and stathmin-specific shRNA.

### Silencing of stathmin decreased deformability of lung adenocarcinoma cells

Transmission electron microscope (TEM) showed that stathmin silencing impeded the formability of pseudopodia of PC-9 cells, and also weakened the deformability of lung adenocarcinoma PC-9 cells, which indicated that overexpression of stathmin promoted the mobility and migration of lung adenocarcinoma cells (Figure 2E).

### Silencing of stathmin suppressed proliferation of lung adenocarcinoma cells

CCK8 analysis was employed to test the influence of shRNA-mediated stathmin silencing on proliferation of PC-9 cells. The results showed that silencing of stathmin significantly reduced proliferation of PC-9 cells (1.08±0.79%) in a time-dependent manner (at 48, 72 and 96) (Figure 2F, P<0.05), as compared with the blank (1.51±1.25%) and NC (1.45±1.19%) groups (p<0.05) (Table 2).

**Table 2:** Assay of proliferation, apoptosis, clone formation, adhesion ability and invasion of cells in vitro

Methods	Cell Proliferation (%)	Apoptosis Rates (%)	Clone Formation (numbers)	Cell Adhesion Rates (%)	Cell Invasion Account (numbers)
Blank	1.51±1.25	2.67±1.53	105.00±7.00	98.28±2.27	60.33±3.76
NC	1.45±1.19	4.33±1.15	110±5.92	96.36±15.62	54.00±4.58
Stathmin-shRNA	1.08±0.79^★^	11.67±2.08^★^	56.33±5.51^★^	57.32±9.53^★^	17.67±1.53^★^
P value	**p<0.05**	**p<0.05**	**p<0.05**	**p<0.05**	**p<0.05**

NC, lung adenocarcinoma PC-9 cells transfected with negative control shRNA; ^★^, PC-9 stathmin- shRNA group compared with PC-9 NC group and PC-9 blank groups.

### Silencing of stathmin induced apoptosis of lung adenocarcinoma cells

**Figure 3:**
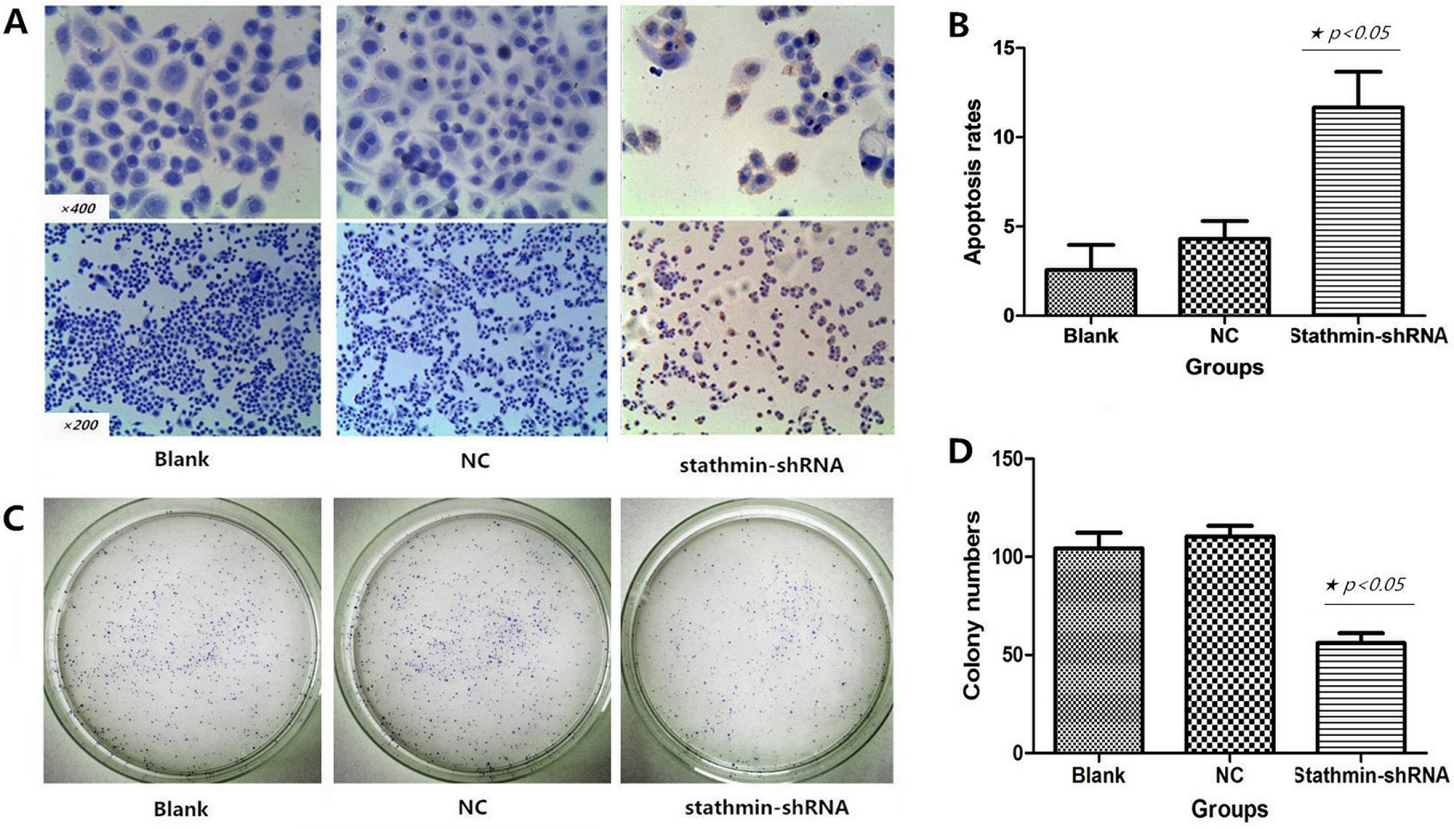
Effects of stathmin-specific shRNA on apoptosis and clone formation of PC-9 cells in vitro **A**. positive cells were found by TUNEL staining: a shrinking cell body, nuclear pyrosis, chromatin condensation and tan or brown granules; **B**. cell apoptosis rate of the stathmin-specific shRNA group was obviously higher than those of the blank and NC groups (p<0.05); **C**. the clone number of stathmin-specific shRNA group was less than blank and NC groups; **D**. the clone number of stathmin-specific shRNA group significantly was decreased, as compared to those of the blank and NC groups (p<0.05); NC, lung adenocarcinoma PC-9 cells transfected with negative control shRNA.

As shown in Figure 3A, TUNEL staining showed that silencing of stathmin induced apoptosis of lung adenocarcinoma cells, which showed following features: chromatic agglutination, karyopyknosis, nuclear fragmentation and brown granules. The apoptosis rate of PC-9 cells transfected with stathmin- shRNA (11.67±2.08%) was remarkably higher than those in blank group (2.67±1.53%) and NC group (4.33±1.15%) (p<0.05) (Figure 3B and Table 2).

### Silencing of stathmin inhibited clone formation of lung adenocarcinoma cells

Plate clone formation assay was employed to evaluate the influence of shRNA-mediated stathmin silencing on proliferation of PC-9 cells. As shown in Figure 3C, the clone number of PC-9 cells transfected with stathmin- shRNA (56.33±5.51) significantly reduced, as compared with those in blank group (105.00±7.00) and NC group (110±5.92) (p<0.05, Figure 3D and Table 2).

### Silencing of stathmin impeded adhesion of lung adenocarcinoma cells

**Figure 4:**
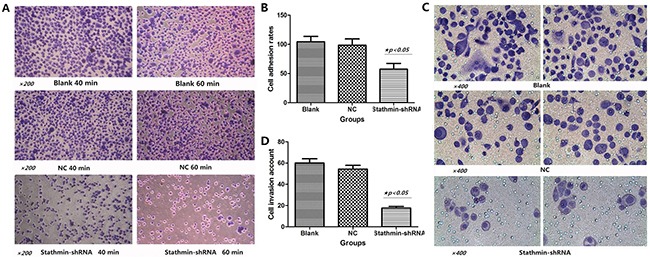
Effects of stathmin-specific shRNA on adhesion and invasion of PC-9 cells in vitro **A**. cell adhesion ability of stathmin- shRNA group significantly reduced in a time-dependent manner (40min and 60min), as compared with the blank and normal control groups; **B**. cell adhesion rate of stathmin- shRNA group was lower than those of the blank and NC groups (p<0.05); **C**. transwell assay revealed that invasion of stathmin- shRNA group significantly reduced compared with the blank and NC groups (p<0.05); **D**. cell invasion number of shRNA group was less than the those of blank and NC groups (p<0.05); NC, lung adenocarcinoma PC-9 cells transfected with negative control shRNA.

As shown in Figure 4A, the cell adhesion of stathmin- shRNA group (57.32±9.53%) significantly reduced in a time-dependent manner (40 and 60 min), as compared with the blank (98.28±2.27%) and NC (96.36±15.62%) groups respectively (Figure 4B and Table 2). This result suggested that overexpression of stathmin promoted the mobility and migration of lung adenocarcinoma cells.

### Silencing of stathmin blocked invasion of lung adenocarcinoma cells

As shown in Figure 4C, Transwell assay revealed that the invasion number of stathmin- shRNA (17.67±1.53) group was remarkably decreased compared with the blank (60.33±3.76) and NC (54.00±4.58) groups (p<0.05) (Figure 4D and Table 2), which suggested that downregulation of stathmin blocked the mobility and migration of lung adenocarcinoma cells.

### Anticarcinogenic efficacy of stathmin silencing may be involved in P38 and MMP2 signal pathways

**Figure 5:**
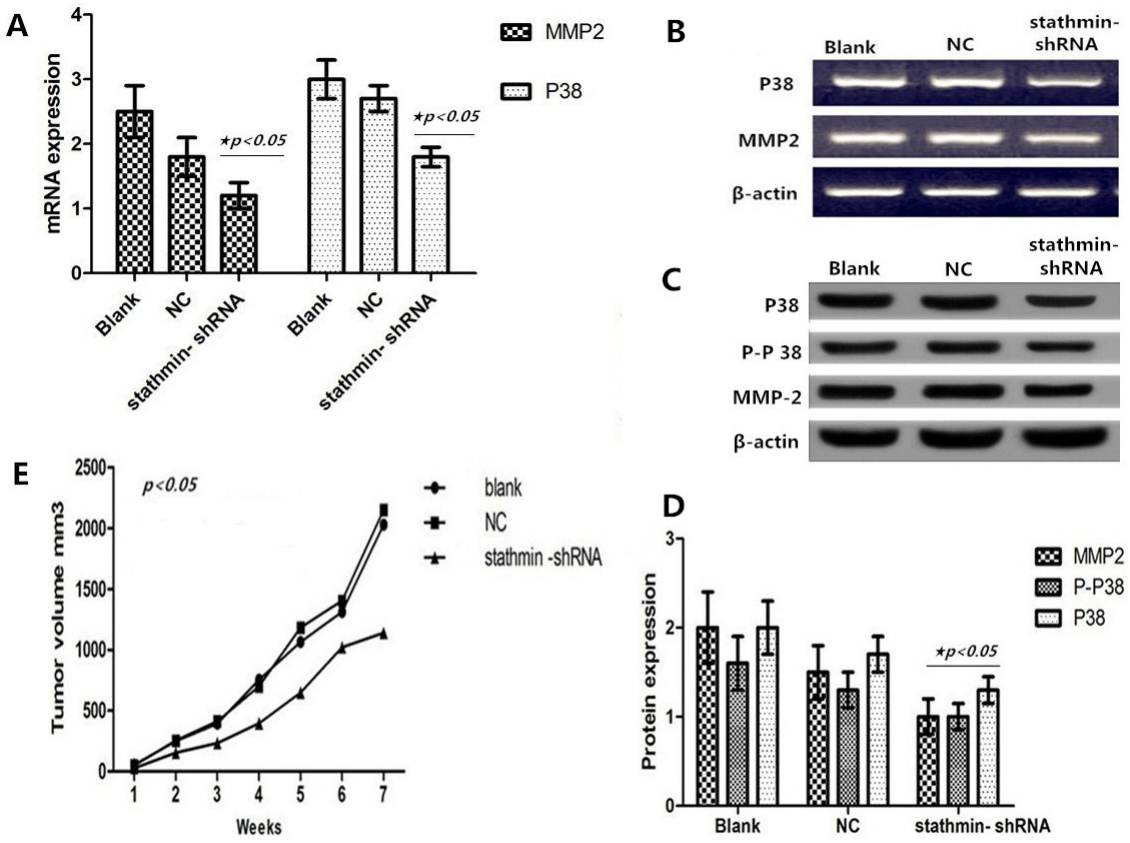
Effect of stathmin silencing on the P38 signally pathway in vitro **A**. silencing of stathmin reduced the mRNA expressions of P38 and MMP2 in PC-9 cells (p<0.05); **B**. mRNA expressions of P38 and MMP2 in RNAi group were less than in blank and NC groups; **C-D**. western blot showed that protein expressions of P38, phosphorylated P38 and MMP2 were downregulated in stathmin- shRNA group compared with the blank and NC groups (p<0.05); **E**. the growth of transplantation tumors in blank and NC groups were relatively quick compared with the stathmin- shRNA group (p<0.05); NC, lung adenocarcinoma PC-9 cells transfected with negative control shRNA.

As shown in Figures 5A and 5B, stathmin silencing led to the mRNA downregulation of P38 and MMP2 in lung adenocarcinoma PC-9 cells (p<0.05). And Western Blot displayed that stathmin silencing downregulated the protein expressions of P38, phosphorylated P38 and MMP2 (Figures 5C and 5D) (p<0.05), suggesting that anticarcinogenic efficacy of stathmin silencing may be involved in P38 and MMP2 signal pathways.

### Silencing of stathmin retarded growth of transplantation tumors

**Figure 6:**
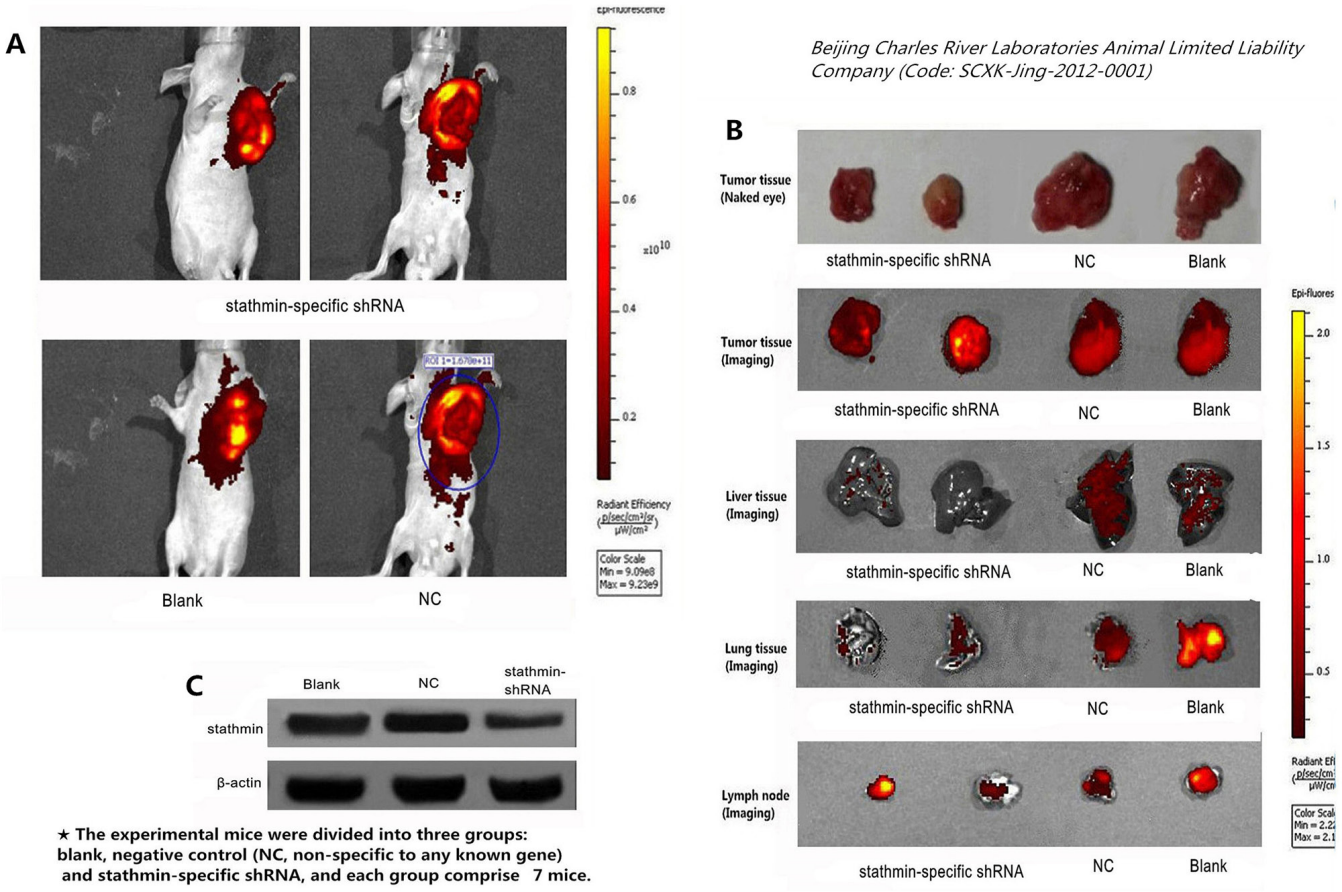
Effect of stathmin silencing on transplantation tumors growth in nude mice **A**. compared with the stathmin-specific shRNA group, the tumors of blank and NC groups had a higher luminescence than those in stathmin-specific shRNA group; **B**. separated tumors from nude mice of stathmin-specific shRNA group were less than those in the blank and NC groups and stathmin silencing prevented metastasis of lung and liver, as well as lymph node metastasis; **C**. stathmin expression in transplantation tumors of stathmin-specific shRNA group was higher than in blank and NC groups (p<0.05); NC, lung adenocarcinoma PC-9 cells transfected with negative control shRNA.

Compared with the blank (2028.33 mm^3^) and NC (2147.9801 mm^3^) groups, the xenografted tumors of the stathmin-specific shRNA group had a slower growth velocity (1137.943 mm^3^) (Table 3 and Figure 5E). In particular, xenografted tumors of the stathmin-specific shRNA group had an earlier burst, ulceration and adjacent diffusion (Figure 6A). In addition, the sizes of xenografted tumors resected from nude mice of stathmin-specific shRNA group were remarkably smaller than those from blank and NC groups, and in vivo imaging of nude mice displayed a consistent trend (Figure 6B). Get together, silencing of stathmin retarded the growth of transplantation tumors in nude mice.

**Table 3:** Growth trend of transplantation tumors in nude mice

Time (weeks)	Overall volume (mm^3^)		
	Blank	NC	Stathmin-shRNA
1	54.10879	49.2356	26.99287
2	249.2315	253.1536	152.9214
3	391.9232	411.4478	233.215
4	753.2844	699.3681	394.3699
5	1062.61	1181.7722	646.8466
6	1312.047	1403.3368	1020.638
7	2028.33	2147.9801	1137.943^★^
Overall	5851.54	6146.294	3634.927^★^
P value	**p<0.05**		

NC, lung adenocarcinoma PC-9 cells transfected with negative control shRNA; ^★^, PC-9 stathmin-shRNA group compared with PC-9 NC and blank groups.

### Silencing of stathmin suppressed metastasis of transplantation tumors in nude mice

The xenografted tumors from PC-9 cells appeared liver and lung metastasis of nude mice in our study. Imaging detecting showed that the lung and liver of stathmin-specific shRNA group displayed an lower luminescence than those in blank and NC groups, suggesting that anti-stathmin treatment prevented metastasis of liver and lung metastasis (Figure 6B). Meanwhile, the expression of stathmin in tumor tissues from the nude mice of stathmin-specific shRNA group was lower than in those in blank and NC groups (p<0.05), thus confirming that this anticarcinogenic efficacy correlated with the silencing of stathmin (Figure 6C).

## DISCUSSION

Stathmin (also known as Op18, p18, p19, or metablastin), a 19-kDa soluble phosphoprotein, is upregulated in some cancers and relates to cell differentiation and proliferation [3–6, 9]. The overexpression of stathmin may activate biological behavior of cancers whereas the inhibition of stathmin expression can interfere with proliferation in cancer cells, inhibiting tumor malignant behavior [4, 9]. A variety of target-specific anti-stathmin investigations have been demonstrated to reduce cell proliferation, clonal growth, cell motility, metastasis and increase apoptosis in malignant tumors [9, 12].

To clarify the stathmin expression in lung cancer, we performed a test of IHC on tissue microarray (TMA). The results showed that 57.9% of LAC presented a higher expression of stathmin, which indicated that overexpression of stathmin may be particularly related to the malignant behavior of LAC. We also noticed that high expression of stathmin was positively associated with poorly differentiated, lymph node invasion and advanced TNM stage in LAC, which suggested that stathmin has the potential to be a prognostic marker for LAC. Our findings are consistent with previous some investigations, where overexpression of stathmin in cancers is associated with a poor prognosis [5, 9, 12–15]. Some studies reported that abnormal expression of stathmin could lead to the dysfunction of microtubule assembly and disorder of cell cycles regulation [9, 12, 14, 15]. And, stathmin has already been found to be involved in the oncogenesis of a wide variety of human cancers [3–5, 7, 9, 10, 12, 15, 16]. Thus, this is possibility that overexpression of stathmin may potentially promote oncogenesis and development of LAC, which contributes to cell proliferation, motility and metastasis of LAC.

We found that stathmin silencing of LAC PC-9 cells transfected with stathmin-specific shRNA significantly surpressed the proliferation, adhesive and invasiveness of PC-9 cells, indicated that overexpression of stathmin promoted the proliferation, migration and invasion of LAC. Previously, a variety of target-specific anti-stathmin investigations have been demonstrated to reduce cell proliferation, clonal growth, cell motility and metastasis [4, 17, 18]. Several studies suggest that there is a intimate correlation between stathmin expression and cell-cycle regulation. Knockdown of stathmin lead to cell cycle arrest in G2/M phase, thus reduces the viability, colony formation and cell proliferation [9, 12, 15, 19]. Stathmin is the important member of a family of microtubule-destabilizing proteins [20], has been showed participating the transcriptional and post-transcriptional regulation of cell cycle progression [9]. We observed that silencing of stathmin induced apoptosis of PC-9 cells, which suggested that stathmin overexpression could increase the progression of LAC via apoptosis inhibition mechanism. Previous studies demonstrate that stathmin present an anti-apoptotic activity to prompt the progress of tumor cells and play an important role in the control of cell cycle [17, 18, 21–23]. Excitedly, we also found that pseudopodia of PC-9 cells transfected with stathmin-specific shRNA significantly reduced and cell deformation ability decreased, expounding that treatment of anti-stathmin could suppress cell movement and invation. Supporting evidence also reports in gastic cancer, in which movement invasion of cells was effectively suppressed by stathmin silencing in the Matrigel invasion assay [10].

Previous investigation shows that stathmin not only plays an important role in cell proliferation and motility but also participates as a “relay protein” in several intracellular cancer related signaling pathways, such as the Hedgehog signaling pathway [24], p53 pathway [16] and Pak1-WAVE2-kinesin complex [25]. Although many studies have identified that stathmin is involved in the activation and inactivation of motility in tumor pathological process [6, 12, 15–18, 23], much still has not be disclosed pertaining the molecular signally pathways how stathmin performs the role. We found that silencing of stathmin incurred the reduction of the relevant proteins of P38-MAPK signal pathway including P38, phosphorylated P38 and MMP2, suggesting that the anticarcinogenic efficacy of stathmin silencing might be involved in P38 signaling pathways. One study has identified stathmin as a specific target of apoptosis signal-regulating kinase 1 (ASK1)-p38 cascade [26], and stathmin can increase cell migration via cooperative activation of p38 [27]. Overexpression of MMP2 has been found to be involved in the tumorigenesis, progression and prognosis of some cancers [28, 29]. Our findings showed that stathmin silencing decreased expression of MMP2, suggesting that stathmin might promote the proliferation and invasion of LAC via upregulating the expression of MMP2.

The anticarcinogenic efficacy of stathmin knockdown on the growth and metastasis of implanted cancer in nude mice have been proved in some previous studies [4, 9, 10, 30]. We demonstrated that not only the volumes of transplantation tumors and the numbers of lung and liver metastatic reduced in the nude mice after stathmin-shRNA treatment, but lymphatic metastasis also significantly abated, which strongly suggested that silencing of stathmin exerted the anti-proliferation and anti-metastasis effect on LAC. Nowadays, many kinds of target-specific anti-stathmin effectors, including ribozymes, monoclonal antibody, shRNA and siRNA have been used extensively to decrease the expression of stathmin in vitro and vivo to investigate the therapeutic strategies targeted towards stathmin [4, 7, 9, 10, 12, 15, 23, 30, 31]. In human cancer, stathmin is usually over-expressed, and decrease the expression of stathmin in vitro and vivo always significantly reduce cell proliferation, clonal growth, cell motility, metastasis and increased apoptosis [4, 7, 9, 10, 12, 15, 30]. Together, it is very possible that an‘anti-stathmin’ targeted therapy could provide a reliable strategy to specifically impede tumor proliferation, motility, migration and occurence of metastasis.

## MATERIALS AND METHODS

### Culture of cell lines

Lung adenocarcinoma PC-9 cells were purchased from Fuxiang Cell Biology Limited Company, Shanghai, China. The cells were cultured in an RPMI 1640 medium, which included 10% FBS, 100 units/mL penicillin, and 100 μg/mL streptomycin (Invitrogen). We maintained the culture solution at 37°C with a humidified 5% CO_2_ and 95% air incubator. When the cells grew into a logarithmic phase, we harvested the cells and carried out a series of following investigations.

### BALB/c nude mice

Twenty-eight female BALB/c nude mice aged 4-6 weeks obtained from Beijing Charles River Laboratories Animal Limited Liability Company (Code: SCXK-Jing-2012-0001). The nude mice were fed and raised separately under specific-pathogen free (SPF) conditions in the Centre of Animal Studies of Xi'an Jiaotong University. We strictly controlled the living conditions as follows: temperature ranged from 20 to 22°C; humidity ranged from 40% to 60%; and artificial illumination was regulated as a light/dark cycle per 12h. The Beijing Medical Experimental Animal Care Commission approved the experimental protocol and followed by institutional review board guidelines.

### Patients

We collected 80 lung cancer tissues from primary lung cancer patients who underwent surgical resection of tumors at the Second Affiliated Hospital, Xi'an Jiaotong University, Shaanxi, China. We obtained written and informed consent individually from all the patients. The study was approved by the Research Ethics Committee of Second Affiliated Hospital, Xi'an Jiaotong University, Shaanxi, China. The patients with lung cancer were divided into different groups according to different clinical features [32] (Table 1). In addition, we also collected 80 matched adjacent normal tissues as the normal control, which were separated from the edge of tumor mass at least 3 cm away.

### Pathological study and tissue microarray construction

In order to construct TMA, we invited two pathologists to discern and mark representative tumor areas. TMA was constructed according to a method previously described [33, 34] using Manual Tissue Arrayer (Beecher Instruments, USA). The constructed TMA block was sliced and kept in refrigerator at 4°C, and further tested with H&E staining and IHC.

### IHC

IHC was employed to determine the expression of stathmin in tissues (S-P Kit, product code: SP9000, Zhongshan Jinqiao Biotech Company, Beijing, China) according to a method previously described [33] (anti- stathmin antibody, 1:200 dilution, Abcam Company). The positive control was stathmin-positive slices provided by biological company, and PBS as a replacement was used as the negative internal control. Two pathologists blindly evaluated the immunostaining scores according to a method previously described [33].

### Western blot

Western blot was employed to disclose the expression status of proteins according to a method previously described [33] (anti- stathmin 1:800; anti- MMP2 1:1000, Abcam Company; anti- p38 MAPK 1:1000, Abcam Company; Phospho-p38 MAPK Antibody 1:1000, BioVision Inc.). We used β-actin as the internal control.

### Real-time quantitative PCR

We used RNA simple total-RNA kit (Tiangen Biotech, Beijing, China) to isolate total RNA, and also performed a real-time quantitative PCR (RT-PCR) using SYBR Green (Tiangen Biotech, Beijing, China) according to the manufacturer's instructions. The conditions of amplification: pre-incubation at 94°C for 6 min; 38 cycles at 95°C for 5 s; 7°C for 45 s; and 72°C for 30 s. We analysed the quantity of mRNA using the 2^−ΔΔCt^ method and β-actin served as the internal control. Primer sequences of mRNA for detection targets in this study are as follows: β-actin forward 5’-ATCATGTTTGAGACCTTCAACA-3’; reverse 5’-CATCTCTTGCTCGAAGTCCA-3’; stathmin forward 5’-GAGAAACGAGAGCACGA-3’; reverse 5’-ATTTAGGAAGGGGATGG-3’; P38 forward 5’-CATTACTTACATCACATGCTACAAA-3’; reverse 5’-ATG ACAGGGCTCAGCAGACT-3; MMP2 forward 5’-GATG CCGCCTTTAACTGG-3’; reverse 5’-TCAGCAGCCT AGCCAGTCG-3.

### RNA interference treatment of stathmin

We searched for the gene sequences of stathmin from the GeneBank (MedLine, USA) and designed the oligonucleotide sequences using Vector NTI 11.5. And we performed a BLAST search of human genome database to assure that the synthesized oligonucleotide sequences would not target other gene transcripts. Finally, stathmin short hairpin RNA (shRNA) and non-specific shRNA were structured and introduced into cell PC-9 cell lines in Beijing Dingguo Changsheng biotechnology CO.LTD. The constructed sequences usd in this study are as follows: Sense chain: 5’-GATCC-(GN18)-(TTCAAGAGA)-(N18C)-TTTTTTG-3’; Antisense chain: 3’-G(CN18)-(AAGTTCTCT)-(N18G)-AAAAAACTTAA-5’; Si-stathmin: TTATTAACCATTCAAGTCC; Sense chain: GATCCGTTATTAACCATTCAAGTCCTTCAAGAGAGGACTTGAATGGTTAATAACTTTTTTG; antisense chain: AATTCAAAAAAGTTATTAACCATTCAAGTCCTCTCTTGAAGGACTTGAATGGTTAATAACG; NC: 5’-TTCTCCGAACGTGTCACGT-3’ 5'GATCCGTTCTCCGAACGTGTCACGTTTCAAGAGAACGTGACACGTTCGGAGAACTTTTTTG-3’ 5'AATTCAAAAAAGTTCTCCGAACGTGTCACGTTCTCTTGAAACGTGACACGTTCGGAGAACG-3’.

### Cell transfection

We used Lipofectamine 2000 (Invitrogen) to do the transfection of shRNA according to the manufacturer's instructions. Briefly, the PC-9 cells were seeded at a concentration of 2×10^5^ cells/well in a six-well plate and incubated until ∼80% confluence. Then, the Lipofectamine 2000 (Invitrogen) and shRNA were mixed gently and incubated together in Opti-MEM I at room temperature for 20 min. Subsequently, the PC-9 cells were incubated in the shRNA-Lipofectamine complex-containing medium for 48 h, and then harvested for further assays. The experiments were divided into three groups: blank, negative control (NC, non-specific to any known gene), and stathmin-specific shRNA.

### Transmission electron microscopy (TEM)

Briefly, we washed PC-9 cells with PBS for twice, and added 25 ml/l glutaraldehyde to fix cells for 30 min. Then, we used 10 g/l osmic acid to fix the cells for 1h. Subsequently, we placed the cells in acetone for gradient dehydration and for displacement in embedding medium (1:1) for 30 min. After 2h in embedding reagents, we observed morphology and structure of cells under TEM.

### Cell proliferation assay

We employed the Cell Counting Kit-8 solution to perform proliferation assay of cells according to manufacturer's instructions. Briefly, PC-9 cells were seeded in 96-well culture plates at a density of 1×10^5^ cells/100 ml/well, and then added 10 ml/well of the Cell counting Kit-8 solution and incubated for 4 h [5]. After that, we used a microplate reader to measure the optical density of the well.

### TUNEL staining

When PC-9 cells grew on glass coverslips, we used 4% paraformaldehyde to fix the cells at least for 30 min. Next, we added 50ul of 0.1% Tritons X-100 to the cells and incubated at room temperature for 20 min. Then the cells were treated using TUNEL kit (11684817910, Roche) according to the manufacturer's instructions. We adopted the following formula to calculate the apoptotic index: apoptotic index = (total number of apoptotic cells/total number of cells) ×100%.

### Colony formation assay

Briefly, 0.5% agar (Sigma) was added in 60 mm dishes, then the treated PC-9 cells were digested into single cell suspension. PC-9 cells were mixed with 0.3% soft agar and added on the bottom agar with 2×10^3^ cells per dish respectively. After that, we observed the cell clone formation under microscope. It was advisable that the cell clone formation was seen but not connected. Subsequently, we washed the cells twice with PBS, fixed with 4% paraformaldehyde for 20 min, and stained with Giemsa solution for 15 min. We counted the number of colonies under microscope.

### Matrigel invasion assay

Briefly, Matrigel basement membrane (BD Biosciences, USA) were dissolved in the ice bath overnight. Next day, the matrigel basement membrane were diluted by MEM 1:100. And 300ul of matrigel basement membrane were added to the each well and embedded overnight. After that, PC-9 cells were trypsinized into single cell suspension, and were seeded in 24-well culture plates at a density of 1×10^5^ cells/well. Then we observed the cells at 20, 40 and 60min, and removed the non-adherent cells respectively. Subsequently, we washed the cells twice with PBS, fixed with 4% paraformaldehyde for 20 min, and stained with Giemsa solution for 15 min, then counted the adherent rate and photographed.

### Cell migration assay

Briefly, PC-9 cells were trypsinized into single cell suspension, and were seeded in a 24-well transwell chamber (3428, Corning, NY, USA) at a density of 1×10^5^ cells/well, which were incubated for 48h at 37°C in constant temperature and humidity incubator. After that, transmemberane were fixed with 4% paraformaldehyde for 20 min, and were washed for 3 times with PBS. Subsequently, we added 50ul of 0.1% Tritons X-100 to the cells, and counterstained with hematoxylin.

### Establishment of the transplantation tumor in node mice

The 28 nude mice models were divided into three experimental groups: blank, NC and stathmin- shRNA. The lung adenocarcinoma PC-9 cells were transplanted via subcutaneous injection of 5×10^7^ cells into the left flank of each nude mouse respectively. One week after transplantation, tumors had grown to a volume of approximately 20 mm^3^, which indicated that the model establishment succeeded. Eventually, a total of 21 nude mice (7 nude mice each group) are qualified to be included for statistical analysis.

### Calculation and observation of transplantation tumor

The volume and size of a tumor was measured calculated according to a calculated standard previously described [19]. The computing formula is as follows: tumor volume (mm^3^) V=(a2×b)/2, where a=the shortest diameter and b=the longest diameter of the tumor (in mm), forming the tumorgrowth curve.

### Vivo imaging observation of model mice

Two mongths after inoculation, the situation of tumor growth and metastasis were searched in node mice by non-invasive optical imaging that was activated by tumor-specific luciferase. Briefly, experimental mice were injected by luciferase substrate luciferin (150 mg/kg) while anesthetized by 2% isoflurane. The signal intensity on transplantation tumor of nude mice was detected using Living Image 4.0 (PerkinElmer, Alameda, CA, USA). The concrete quantity at the selected region of interest covering the tumor were identified by peak photon flux.

### Statistical analysis

We used SPSS 22.0 software package (SPSS Institute, Chicago, USA) to make a statistical analysis. The enumeration data pertaining to stathmin expression and clinico-pathological features were calculated using the χ^2^ and Fisher's exact test. The data belonged to continual variables (mean ± SEM) was analysed using the Student's *T*-test, and One–WAY ANOVA Test. All tests were two-sided, and p-values <0.05 were considered to be statistically significant.
